# Intra-islet lesions and lobular variations in β-cell mass expansion in *ob*/*ob* mice revealed by 3D imaging of intact pancreas

**DOI:** 10.1038/srep34885

**Published:** 2016-10-07

**Authors:** Saba Parween, Elena Kostromina, Christoffer Nord, Maria Eriksson, Per Lindström, Ulf Ahlgren

**Affiliations:** 1 Umeå Centre for Molecular Medicine, Umeå University, Umeå, Sweden; 2 Integrative Medical Biology, Umeå University, Umeå, Sweden

## Abstract

The leptin deficient *ob*/*ob* mouse is a widely used model for studies on initial aspects of metabolic disturbances leading to type 2 diabetes, including insulin resistance and obesity. Although it is generally accepted that *ob*/*ob* mice display a dramatic increase in β-cell mass to compensate for increased insulin demand, the spatial and quantitative dynamics of β-cell mass distribution in this model has not been assessed by modern optical 3D imaging techniques. We applied optical projection tomography and ultramicroscopy imaging to extract information about individual islet β-cell volumes throughout the volume of *ob*/*ob* pancreas between 4 and 52 weeks of age. Our data show that cystic lesions constitute a significant volume of the hyperplastic *ob*/*ob* islets. We propose that these lesions are formed by a mechanism involving extravasation of red blood cells/plasma due to increased islet vessel blood flow and vessel instability. Further, our data indicate that the primary lobular compartments of the *ob*/*ob* pancreas have different potentials for expanding their β-cell population. Unawareness of the characteristics of β-cell expansion in *ob*/*ob* mice presented in this report may significantly influence *ex vivo* and *in vivo* assessments of this model in studies of β-cell adaptation and function.

The pancreas is a mixed endocrine and exocrine gland that plays a pivotal role in maintaining blood glucose homeostasis. The endocrine component, organized into the islets of Langerhans, constitute a fraction of the total pancreatic mass (≈1–2%) and is scattered throughout the volume of the gland^1^. The most prominent endocrine cell type is the insulin producing β-cell, which in mice are located in the core of the islets. In response to an increased demand for insulin, the pancreatic β-cells may adapt by augmenting insulin secretion either via increased β-cell function, increased β-cell mass (BCM), or both. It is well established that the leptin deficient *ob*/*ob* mouse exhibits both of these features. Since it was first described in 1950 2, the strain has been extensively studied as a model for type 2 diabetes (T2D), including aspects of insulin resistance and obesity, and the marked expansion of BCM has made it a rich source of β-cells for studies of islet function in numerous publications (see refs 3 and 4 and references therein).

A detailed understanding of BCM dynamics is a key element to fully appreciate the pathophysiology of models of diabetes and metabolic stress. Whereas it has been demonstrated that BCM distribution may vary significantly between strains of different genetic background^5^, few studies exist that addresses the dynamics of BCM distribution within the entire pancreas, be it in normal mice or in disease models. One of the most comprehensive stereological assessments of BCM in the *ob*/*ob* mice determined that BCM expansion was primarily due to expansion of already existing islets^6^. Another study, using bioluminescence imaging (BLI) to monitor BCM in *ob*/*ob* mice *in vivo*, also incorporated an extensive stereological β-cell quantification of the model^7^. Although these and other studies provided important information on the BCM dynamics of *ob*/*ob* mice, they depend on the extrapolation of two-dimensional data (as do all stereological approaches) or, as in the case of BLI, provide only diminutive spatial information. Whereas it has been demonstrated in other models for insulin resistance that β-cell adaptation could be topologically heterogeneous^8^, no information of this kind exists for the *ob*/*ob* model.

Previously, we developed an optical projection tomography (OPT)^9^ based approach that enables detailed extraction of the islet distribution throughout the volume of the intact pancreas^10^,^11^. Hereby, individual islet β-cell volumes and their spatial 3D coordinates may be derived in large cohorts of animals^12^,^13^,^14^. In this study we used OPT imaging to describe the development of islet hypertrophy in the *ob*/*ob* mouse between 4 and 52 weeks of age. We provide detailed statistics on BCM expansion (to better adhere with the principle of detection: β-cell volume (BCV) will be used throughout) and its topological distribution, as well as a putative mechanism for the formation of a cystic aberration forming in hyperplastic *ob*/*ob* islets.

## Results

### The lobular compartments of the pancreas in *ob*/*ob* mice display different capacities for BCV expansion

**Figure 1:**
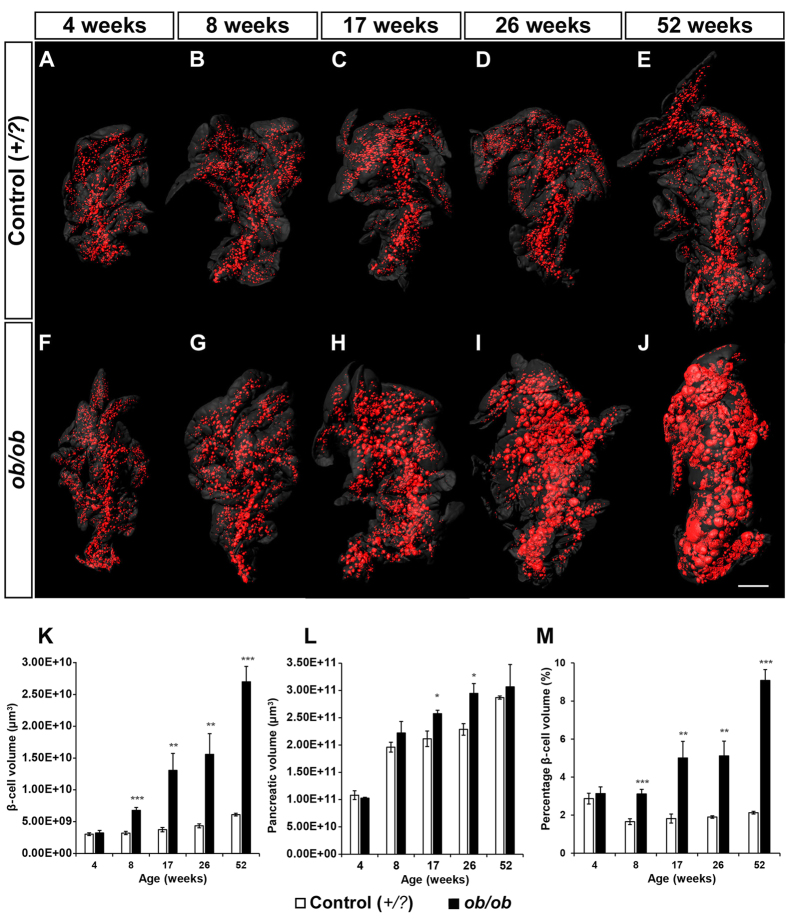
Islet β-cell distribution in *ob*/*ob* and lean control mice between 4 and 52 weeks of age. (**A**–**J**) Iso-surface rendered OPT images of representative splenic lobes of lean control (**A**–**E**) and *ob*/*ob* (**F**–**J**) pancreata. The islet **β**-cell volumes are reconstructed based on the signal from insulin specific antibody staining (red) and pancreas outline (gray) is based on the signal from tissue autofluorescense. In contrast to lean controls, the expected expansion in BCV is clearly observed in *ob*/*ob* pancreata. (**K**) Graph showing the average total β-cell volume in *ob*/*ob* and lean controls illustrating the progressive increase in *ob*/*ob* BCV (entire pancreas). (**L**) Graph showing the average total pancreas volume. (**M**) Percentage of the total pancreatic volume constituted by insulin positive cells. Data is shown as means ± SEM (n = 5) where *P < 0.05; **P < 0.01 and ***P < 0.001. Scale bar in (**J**) corresponds to 2 mm in (**A–J**).

**Figure 2:**
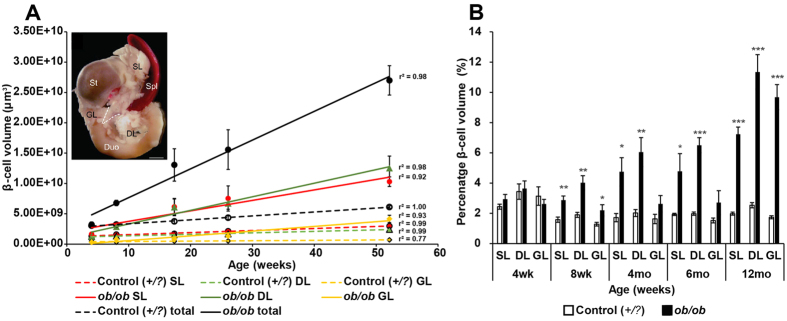
Lobular pancreatic β-cell volumes in *ob*/*ob* and lean controls. (**A**) Graph illustrating the total average (black lines) and lobular splenic (SL, red), duodenal (DL, green) and gastric (GL, yellow) β-cell volumes in lean control (broken lines) and *ob*/*ob* pancreata (intact lines). The BCV expansion is close to linear in all groups (r^2^ scores are indicated). (**B**) Graph displaying the average percentual β-cell volume in the respective splenic, duodenal and gastric lobes of lean control (white bars) and *ob*/*ob* (black bars). Inset shows a photomicrograph of a gut segment including the stomach, duodenum, spleen and pancreas from a C57Bl/6 mouse at 8 weeks, illustrating the delineation of the lobes. Data is shown as means ± SEM (n = 5) where *P < 0.05; **P < 0.01 and ***P < 0.001. Abbreviations: SL, Splenic lobe; DL, Duodenal lobe; GL, Gastric lobe; St, Stomach; Spl, Spleen; Duo, Duodenum. Scale bar in inset is 2 mm.

In order to refine our current information regarding the quantitative dynamics of β-cell hyperplasia and to generate a first 3D-spatial account on islet hypertrophy in the *ob*/*ob* mice, we subjected intact pancreatic lobes from *ob*/*ob*^*Umeå*^ mice and lean (+/+ *or* +/*ob*) littermates (referred to as +/*?*) at 4, 8, 17, 26 and 52 weeks of age to OPT imaging. As expected we could both visually and quantitatively observe a gradual increase in pancreatic BCV over time (Fig. 1A–J (splenic lobes), Fig. S1 (duodenal lobes) and Fig. S2 (gastric lobes). Whereas no significant differences could be detected in total BCV between *ob*/*ob* and controls at 4 weeks of age, the average total BCV in the *ob*/*ob* mice were 2.1, 3.5, 3.6 and 4.4 times higher *in ob*/*ob* pancreata compared to control between 8 and 52 weeks respectively (Fig. 1K). At these stages, the overall volume of the gland was slightly enlarged (Fig. 1L). The BCV expansion in *ob*/*ob* pancreata was close to linear (R^2^ = 0.98) and increased from around 3% of the total pancreas volume at 4 weeks to around 9% at 52 weeks (Figs 1M and 2A). A close to linear BCV expansion was observed also when assessing the individual splenic, duodenal and gastric lobes (R^2^ = 0.98, 0.92 and 0.93 respectively) (Fig. 2A). Similar to normal C57Bl/6 mice^1^, the splenic lobe of *ob*/*ob* mice harbored a higher BCV than the duodenal and gastric lobe at early stages, i.e. 4 and 8 weeks of age. The same pattern was observed for lean control mice until 52 weeks of age. However, the relative BCV expansion differed between the primary lobular compartments in *ob*/*ob* mice. Between 4 and 52 weeks of age, the gastric lobe increased its BCV 13.7 times, the duodenal lobe 9.8 times and the splenic lobe 6.3 times. In contrast to lean control mice, the average BCV of the *ob*/*ob* duodenal lobe even surpassed that of the *ob*/*ob* splenic lobe (Fig. 2A). Notably, the most dramatic increase in BCV in the gastric lobe occurred between 26 and 52 weeks. In comparison, the lobes of lean control mice increased their BCV 1.46, 1.93 and 2.29 times for the gastric, duodenal and splenic lobes respectively during the same time frame. Lobular heterogeneities in BCV distribution were also observed in terms of β-cell densities. Being very similar to lean controls at 4 weeks of age, the *ob*/*ob* BCV constituted 7.2% (splenic), 9.7% (gastric) and 11.4% (duodenal) of the overall lobular volumes at 52 weeks of age (see Fig. 2B). Noteworthy, the duodenal lobe displayed the highest β-cell density throughout the investigated stages, both in *ob*/*ob* and lean control pancreas (Fig. 2B). Our data further suggests that the BCV continues to expand throughout the investigated stages. Staining with the cell proliferation marker Ki67 indicated that the rate of β-cell proliferation is prominent in *ob*/*ob* mice also at the end point of our investigation (Fig. S3). Jointly, these results show that islet hypertrophy is characterized by a close to linear BCV expansion in all three primary lobes of the pancreas within the investigated time span. Further, they indicate that the primary lobular compartments of the pancreas have varying capacities to expand their β-cell population in response to increased insulin demand in *ob*/*ob* mice.

### β-cell hyperplasia occurs in islets of all size categories

**Figure 3:**
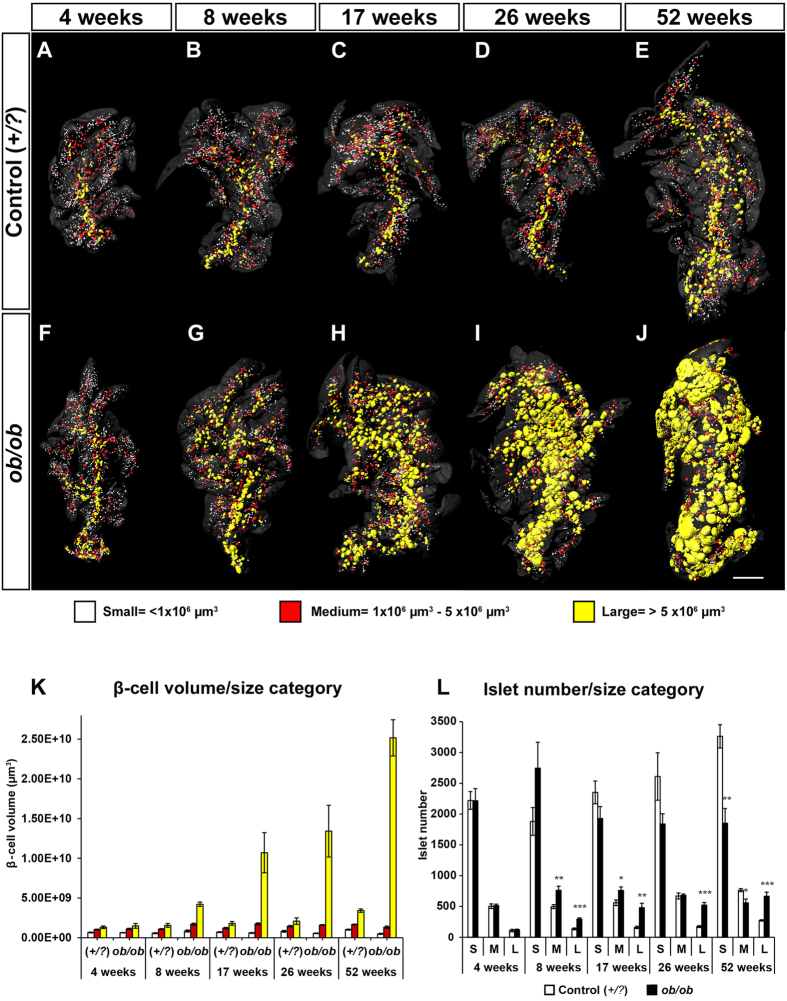
o*b*/*ob* β-cell expansion by islet β-cell volume size categories. (**A**–**J)** Iso-surface rendered OPT images of representative splenic lobes of lean control (**A**–**E**) and *ob*/*ob* (**F–J**) pancreata between 4 and 52 weeks of age. Individual islet **β**-cell volumes are reconstructed based on the signal from insulin-specific antibodies and have been pseudo colored to highlight the distribution of small (<1 × 10^6^ μm^3^ [white]), intermediate (1 × 10^6^ −5 × 10^6 ^μm^3^ [red]), and large (>5 × 10^6^ μm^3^ [yellow]) islets. (**K)** Histogram showing the average total **β**-cell volume constituted by each size category in lean control and *ob*/*ob* pancreata respectively. (**L**) Histogram showing the average total number of islets within each size category in lean control and *ob*/*ob* pancreata respectively. Data is shown as means ± SEM (n = 5) where *P < 0.05; **P < 0.01 and ***P < 0.001. Abbreviations: S, small; M, medium; L, large. Scale bar in (**J**) corresponds to 2 mm in (**A**–**J**).

**Figure 4:**
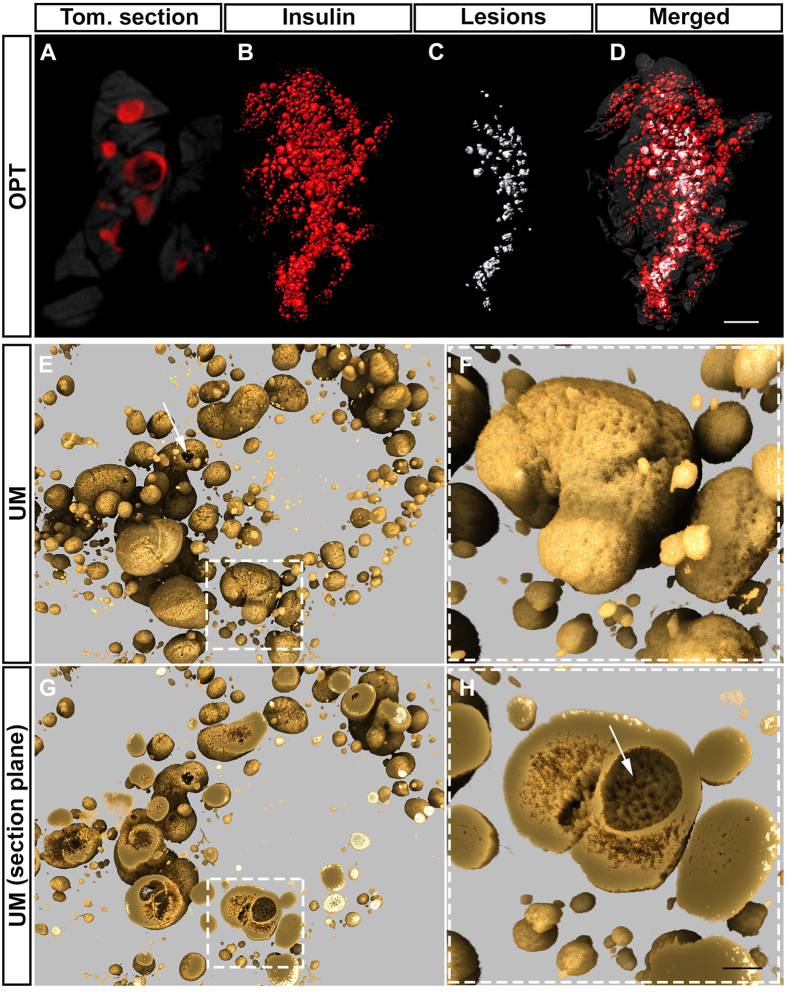
Cystic lesions are frequently formed in hypertrophic *ob*/*ob* islets. (**A**) Tomographic OPT section from a representative *ob*/*ob* pancreas at 26 weeks of age (splenic lobe). Insulin positive areas are pseudocolored red. (See also Movie S2). (**B**–**D**) Iso-surface rendered OPT images of the specimen in (**A**). The islet **β**-cell volumes are reconstructed based on the signal from insulin specific antibody staining (**B**, red) and the outlines of the cystic lesions were manually delineated in the tomographic sections (**C**, gray). The islet **β**-cell volumes and the areas occupied by lesions are merged together with the outline of the organ (dark gray) in (**D**). (**E**–**H**) Representative ultramicroscopy images of hypertrophic islets at 26 weeks (duodenal lobe) showing the surface morphology (**E**,**F**) and the same islets applying a digital section plane to visualize the internal lesions (**G**,**H**) Area marked by a broken line in (**E**,**G**) corresponds to high magnification images in (**F**,**H**). White arrow in (**E**) points to a cystic lesion that has generated an opening towards the surface of the islet. Abbreviations; SL, Splenic lobe; DL, Duodenal lobe; Tom. section, Tomographic section. Scale bar in (**D)** is 506 μm in (**A**) and 2 mm in (**B**–**D**). Scale bar in H is 291 μm in (**E**,**G**) and 100 μm in (**F**,**G**).

Islets of *ob*/*ob* mice are renowned for their high insulin secreting capacity^3^, and it has been suggested that islets generally display functional differences related to their size. For example, it has been demonstrated that “small” islets contain more insulin per islet volume (*in situ*) and secrete insulin more efficiently (*in vitro*)^15^. Whereas it is evident from our OPT measurements and other studies (ref. 3 and references therein) that the BCV is dramatically increased in the *ob*/*ob* pancreas, it is unclear if islets of different sizes have a similar capacity to expand their β-cell population. Against this background we divided the individual islet β-cell volumes into arbitrarily chosen, small (<1 × 10^6 ^μm^3^), intermediate (1 × 10^6^ μm^3^ −5 × 10^6^ μm^3^) and large (>5 × 10^6^ μm^3^), size categories (Fig. 3). In healthy mice the largest islets are predominantly located centrally in the pancreas whereas smaller islets preferentially appear dispersed peripherally in the lobules^10^,^16^. By cross- referencing pseudo colored size categories back to the 3D images of islet BCV distributions, this pattern could be observed for lean control mice at all ages (Fig. 3A–E). In contrast, *ob*/*ob* mice initially displayed a similar distribution, but with age islets falling within the large size category gradually occupied also the peripheral regions of the gland (Fig. 3F–J). This pattern was confirmed by analyzing the number of islets falling within each size category (Fig. 3K,L). With the exception of 8 weeks, the number of islets falling within the “small” size category, gradually decreased, whereas the number of islets falling within the “large” category increased. The noticeable, but statistically insignificant, relative increase in small islets at 8 weeks in *ob*/*ob* mice was unsuspected since it could indicate *de novo* establishment of islets. However, in order to avoid inclusion of general noise during the reconstruction process, we routinely exclude objects of 10 voxels or less from the iso-surfaced 3D data sets (see methods). Hence it is likely that this increase in small islets at 8 weeks corresponds to β-cell hyperplasia in very small islets, a few β-cells in diameter or even by individual β-cells, making these β-cell volumes growing into detection range. At later stages the picture is complicated by that islet hypertrophy results in a gradual fusion of islets (see Fig. 4), which is likely to reduce the number of counted islets volumes, in particular for the large category. Notwithstanding the above challenges for islet counting, our data indicate that β-cell hyperplasia occurs in islets of all size categories in *ob*/*ob* mice.

### Hypertrophic islets of *ob*/*ob* mice develop internal lesions, which significantly contribute to the overall islet surface area

**Figure 5:**
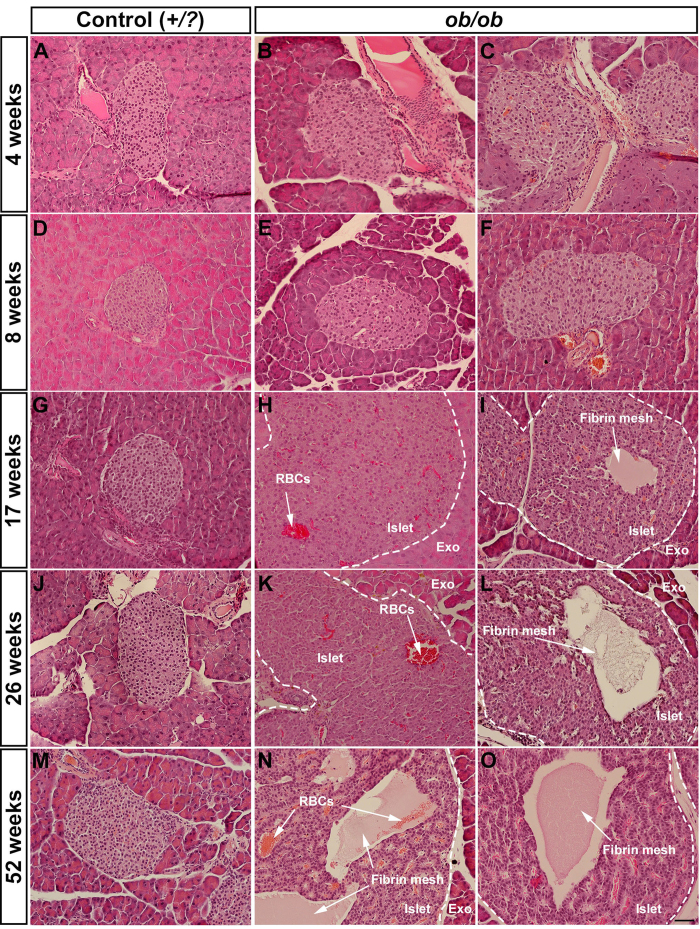
Histological analysis of *ob*/*ob* and lean control pancreata between 4 and 52 weeks of age. (**A**–**O**) Hematoxylin/Eosin staining of representative sections from lean control (**A,D,G,J,M**) and *ob*/*ob* (**B**,**C**,**E**,**F**,**H**,**I**,**K**,**L**,**N**,**O**) at 4 weeks (**A**–**C**), 8 weeks (**D**–**F**), 17 weeks (**G**–**I**), 26 weeks (**J**–**L**) and 52 weeks (**M**–**O**). Abbreviations; RBCs, Red blood cells; Exo, Exocrine tissue. Scale bar in (**O**) is 50 μm in (**A**–**O**).

Whereas the morphology and cytoarchitecture of *ob*/*ob* islets have been assessed in a number of studies (see e.g. ref. 17) only one study reported “occasional” central cystic spaces to be present in the core of hypertrophic islets^18^. The nature and mechanism for formation of these lesions is unknown. Analyses of the tomographic OPT data sets from *ob*/*ob* pancreata showed that cystic lesions is a commonplace feature of hypertrophic *ob*/*ob* islets, at least at later stages. Whereas we could detect the occasional presence cystic lesions at week 8, these were not regularly observed in the *ob*/*ob* islets until week 17, and at 26 weeks of age lesions were formed within the absolute majority of the large islets (Fig. 4A, Movie S1 and Fig. 5). As determined by high-resolution ultramicroscopy (UM) imaging, the lesions occupy a significant portion of the islet volumes and may extend to the very perimeter of the islets, creating a cave like entrance into the islet interior (Fig. 4E–H). Iso-surfacing the volume constituted by the cystic lesions (see methods) in splenic lobes from 26 weeks old *ob*/*ob* pancreas showed that the average volume of the cystic lesions constituted as much as 15.3% (SEM = ± 3.65, n = 4) of the volume enclosed by the β-cells and lesions together (Fig. 4B–D and Movie S2). Note, by nature of the tomographic technique employed, the volumes constituted by cystic lesions were not included in the BCVs presented in this report. At no stage could cystic lesions be detected in the exocrine tissue. Collectively, these data demonstrate that the formation of cystic lesions is a characteristic feature of hypertrophic *ob*/*ob* islets and that they may significantly influence the surface area of the affected islets.

### The formation of cystic lesions in *ob*/*ob* islets is associated with extravasation of red blood cells and the formation of fibrin mesh

**Figure 6:**
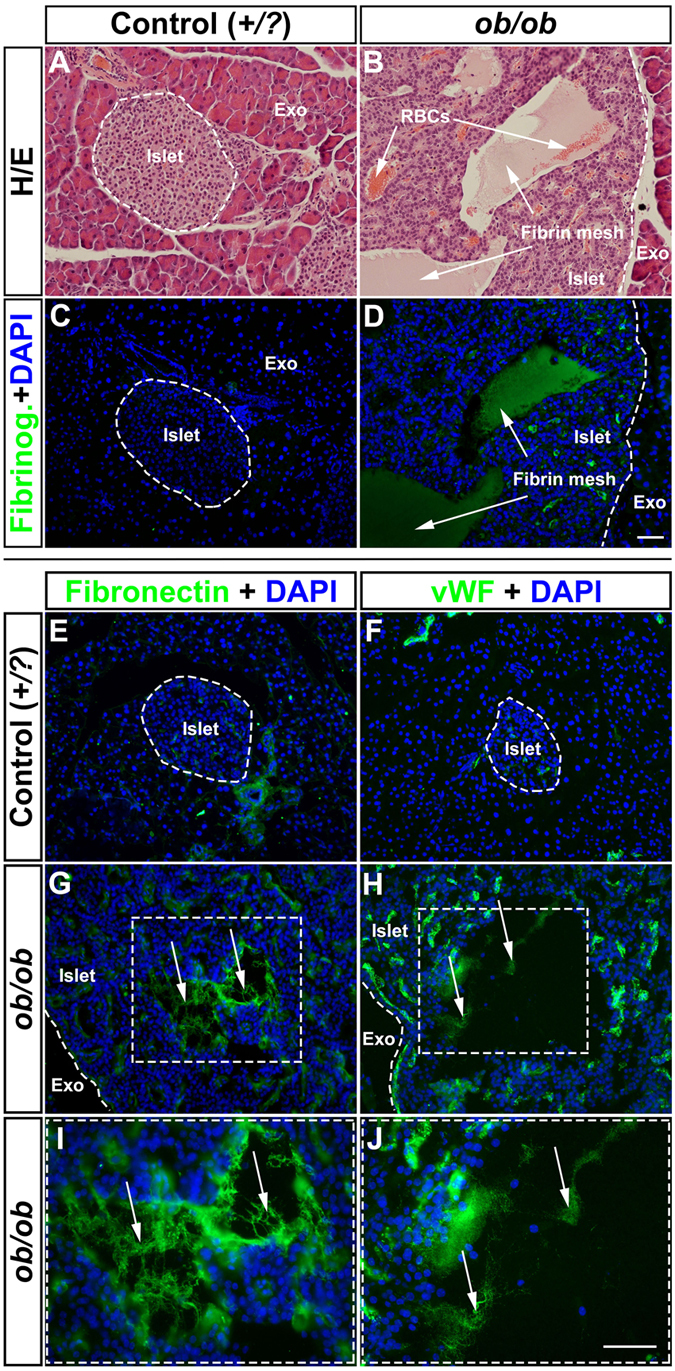
Immunohistochemical assessment of proteins involved in blood coagulation in *ob*/*ob* pancreata. (**A**,**B**) Hematoxylin/Eosin staining of an islet from a lean control (**A**) and a ob/ob (**B**) pancreas at 52 weeks. Note the accumulation of RBCs (white arrows in (**B**). (**C,D**) Consecutive sections to (**A**,**B**) stained for Fibrinogen (green) and DAPI (Blue) indicating the presence of a fibrin mesh within the areas of the lesions (white arrows in (**D**) compare with (**B**)). (**E**–**J)** Photomicrographs of representative pancreatic cryosections from lean control (**E**,**F**) and *ob*/*ob* (**G**,**H**) pancreata at 52 weeks of age labeled for Fibronectin (Green **E**,**G**) and von Willenbrand Factor (Green, **F**,**H**) together with DAPI (blue). Areas enclosed by a broken line in (**G**,**H**) corresponds to (**I**,**J**) respectively. The areas in the lesions positive for Fibronection and von Willenbrand factor are not associated with any nucleated cells. Abbreviations; vWF, von Willenbrand Factor; Exo, Exocrine tisse. Scale bar in (**D**) is 50 μm in (**A**–**D**) and scale bar in (**J**) is 92 μm in (**E**–**H**) and 50 μm in (**I**,**J**).

In order to assess the nature of the cystic lesions of *ob*/*ob* islets, we analyzed histological sections of *ob*/*ob* and +/*?* control pancreata between 4 and 52 weeks. In agreement with the OPT and UM analyses we could detect the formation of lesions in a significant amount of the hypertrophic islets. These appeared to form with a few individual foci in the affected islets (usually one but in some islets several foci could be observed, see Fig. 5). It has previously been reported that islet vessels are dilated in *ob*/*ob* islets^19^. To address the possibility that the lesions reflected an aberration of the intraislet vessels we performed ultramicroscopy imaging of whole mounted pancreata labeled with wheat germ agglutinin lectin. We could determine that the lesions did not represent enlarged or by other means altered vascular structures (Movie S3). Further, by stainings for DAPI, cleaved Caspase 3, TGF-β1 and Hematoxylin/Eosin, we could not detect any signs of nucleated cells, apoptosis or fibrosis, respectively, in the areas of the lesions (Figs 5 and S4). Instead, the histological analyses pointed to the presence of red blood cells (RBCs) and/or a fibrin mesh occupying the lumen of the lesions (see for example Fig. 5N). Whereas smaller lesions primarily appeared to be occupied by RBCs and the larger by fibrin mesh, the relative contribution of these components varied both with lesion size and age (Fig. 5). The majority of the mesh-like structures were positive for fibrinogen (Fig. 6A–D) and to a various extent stained positive for von Willebrand factor and fibronectin, both proteins involved in blood coagulation^20^,^21^ (Fig. 6E–J). Jointly these results point to a role for blood clotting in the formation of the cystic lesions of *ob*/*ob* islets.

## Discussion

Using state of the art techniques for OPT and UM imaging, we provide detailed 3D and quantitative data regarding the BCV expansion in isolated pancreata from *ob*/*ob*^*umeå*^ and lean control mice. While the dynamics of β-cell expansion in *ob*/*ob* mice has been extensively studied by conventional histological techniques our approach provides a more comprehensive picture, including a set of novel insights into this process. Whereas the aggravated growth kinetics of the pancreatic β-cells was expected, the dramatic differences in BCV increase between the primary lobes of the pancreas is not previously reported. The possibility that different islet populations have varying capacities to expand their BCV may have important implications, e.g. in the isolation of islets for grafting purposes in pre-clinical/clinical settings or in attempts of *in vitro* reconstruction of pancreatic islets^22^. It is possible that these differences could be a reflection of the local nature of supporting tissues or interacting cell types such as lobular variations in blood vessel supply or levels of circulating GLP-1. An alternative explanation is that they correlate to more profound differences in the islet populations. Indeed, the pancreatic lobes are formed in different developmental environments and it has been suggested that there are both anatomical (ratios of endocrine cells) and functional differences between islets from the splenic and duodenal portion of the gland^23^,^24^. Our data show that the BCV increase is most dramatic in the gastric lobe, followed by the duodenal and the splenic lobes. We have previously demonstrated that there are striking heterogeneities between the three lobes of the pancreas with regards to islet densities in C5BL/6 mice, with the gastric lobe displaying the highest density^1^. The average size of these islets is however smaller than of those in the duodenal and splenic lobes. Hence, it is possible that the varying capacity for lobular BCV expansion in the *ob*/*ob* mouse instead points to a generally increased capacity for small islets to expand their BCV in response to an increased insulin demand. Assessments of other models for islet hyperplasia, may contribute to shed light on this observation.

Previous studies have reported a decline in *ob*/*ob* BCV between around 30–48 and 50–56 weeks of age^7^,^25^. In contrast, our quantitative OPT data and the immunohistochemical analyses of β-cell proliferation indicate that the BCV in *ob*/*ob* mice is still expanding at the endpoint of our investigation, i.e. at 52 weeks of age. The reason for this discrepancy is unclear. Whereas a normalization of the blood glucose levels have been observed in *ob*/*ob* mice between 30–50 weeks of age^7^,^26^,^27^ our data (of unfasted animals) display a more fluctuating pattern, which is in agreement with previous observations in our and other *ob*/*ob* colonies. I.e., blood glucose values can vary considerably between individual *ob*/*ob* mice in the unfasted state^7^,^26^,^28^,^29^ (see Fig. S5). By nature of the *ex vivo* OPT imaging approach, the animals included in our study were not monitored over time. Instead, the BCV and blood glucose levels correspond to individual animals at the time points for pancreas isolation. A possible explanation for the observed dynamics of BCV expansion in our study is that our data does not incorporate a measurement point between 26 and 52 weeks. However, given the apparent linearity of the BCV expansion in our data set, in combination with the observation of ongoing proliferation at 52 weeks of age, this seems unlikely. Differences in the methodologies utilized (stereology and/or *ex viv*o bioluminescence versus OPT) may further contribute to this discrepancy. Since previous studies assessing BCM dynamics in the *ob*/*ob* mice^7^,^25^ did not report the formation of cystic lesions, it is unclear how this circumstance may have affected BCM quantitations in these studies. Notwithstanding the seemingly high variation in blood glucose levels at later stages, our data raises the possibility that physiological factors other than an increased demand for insulin^30^,^31^ may contribute to BCV expansion in the *ob*/*ob* mouse.

**Figure 7:**
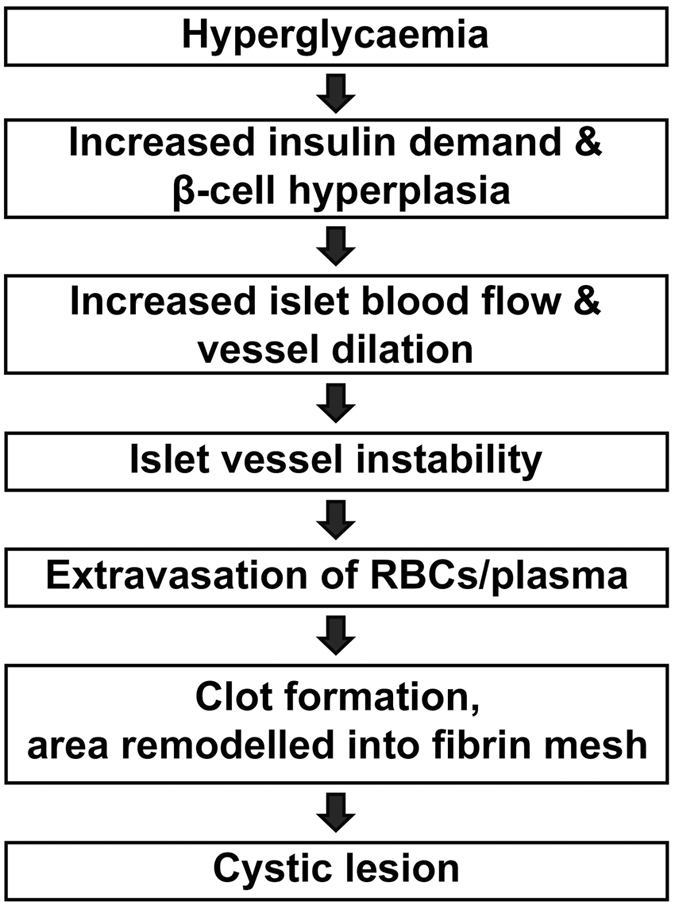
Cystic lesions are formed in *ob*/*ob* islets by a mechanism involving extravasation of red blood cells. Schematic illustration of a putative model for lesion formation in *ob*/*ob* islets (see text for details).

We report that cystic lesions are a commonplace feature of hyperplastic islets in *ob*/*ob* mice. These lesions contribute to a significant increase of the overall islet surface area. At 26 weeks of age the volume of the lesions correlated to the volume constituted by β-cells in the corresponding lobe of lean control mice (*ob*/*ob* splenic lobe lesion volume was 2.10 × 10^9^ μm^3^ and the lean control average BCV was 2.15 × 10^9 ^μm^3^). Given the extensive use of the *ob*/*ob* mouse as a model of obesity and insulin resistance, the extent of this dramatic morphological aberration was unexpected. It has previously been shown that an adaptation of the *ob*/*ob* islets to insulin resistance is characterized by increased islet blood flow and islet vessel dilation in combination with structural alterations of the islet vessels, suggested to lead to islet vessel instability^19^,^25^. As a consequence extravasation of RBCs was observed, indicative of intraislet hemorrhaging, predominantly in large islets of *ob*/*ob* mice at 16 weeks^19^. In view of our results, and these reports, we propose a model for intraislet lesion formation in the *ob*/*ob* mouse (summarized in Fig. 7). In agreement with previous observations, we suggest that an increased demand for insulin, triggered by the hyperglycemic state of the animals, results in increased blood flow, vessel dilation and altered vascular structure. This in turn results in leakage of RBCs/plasma from vessels in the *ob*/*ob* islets. The extravasated RBCs will subsequently clot and the area remodel into a fibrin mesh, thereby creating lesions within the core of the islets. Cystic lesions has also been noted in large islets of autopsy material from human T2D subjects^32^. Similar to our observations, the material inside these human islet lesions did not contain nucleated cells. However, the more precise nature of these structures has to the best of our knowledge not been thoroughly examined and further studies will be required to assess if similar mechanisms for intraislet lesion formation are at play also in settings of human T2D. Although the mechanism and extent by which lesions are formed in *ob*/*ob* mice is previously unreported, researchers performing pioneering studies on the *ob*/*ob* model noted the phenomenon, and it was generally accepted that such islets were prone to deviate from *ob*/*ob* islets lacking lesions. Large islets with visual cystic lesions were therefore excluded from *in vitro* experiments since they responded much less, and more variable, to glucose stimulation (Profs. B. Hellman and J-O. Sehlin, personal communication). Although current technology provides limited possibilities to study the impact of lesion formation on endogenous islet function *in vivo*, this could potentially be addressed by studies of grafted islets, e.g. in the anterior chamber of the eye^33^.

In summary, this report provides a detailed 3D and quantitative outline of the BCV dynamics and islet morphology in the *ob*/*ob* mouse. Jointly, our results may influence the use and interpretation of the *ob*/*ob* mouse model in studies ranging from aspects of β-cell adaptation and/or function to the development of antidiabetogenic drugs or non-invasive imaging approaches. They further emphasize that lobular heterogeneities may affect both quantitative, and potentially also functional, assessments of the pancreas unless the entire volume of the gland is taken into account. Finally, they prompt for further studies of the occurrence and impact of islet lesions in settings of T2D in other models, including humans.

## Materials and Methods

### Animals and organ isolation

All experiments were conducted in accordance with Umeå University guidelines and national legislation. All animal experiments were approved by the animal review board at the Court of Appeal of Northern Norrland in Umeå. Animals were killed by cervical dislocation and pancreata from groups (n = 5) of *ob*/*ob*^Umeå^ mice and lean control (*ob*/+ or +/+) littermates were isolated at 4, 8, 17, 26 and 52 weeks of age. In conjunction with organ isolation, the animals were weighed and blood glucose levels were measured in fed mice using by a OneTouch Ultra^®^ glucometer (LifeScan, USA). The pancreata were fixed in 4% PFA and the primary, splenic, duodenal and gastric lobes^34^ were separated before processing for OPT imaging.

### Optical Projection Tomography

The pancreata were stained for insulin and processed for OPT imaging as previously described^10^,^12^. Antibodies used were Guinea Pig anti-insulin (DAKO A0564 1:500) and Goat Alexa 594 anti-Guinea Pig (Molecular Probes A11076). OPT scanning was performed as described using a Bioptonics 3001 OPT scanner (SkyScan, Belgium), applying a contrast limited adaptive histogram (CLAHE) algorithm with a tile size 64 × 64 to the projection images^1^,^11^. Tomographic reconstruction was performed using the NRecon v1.6.9.18 software (Skyscan, Belgium). Insulin positive β-cell volumes were quantified by 3D iso-surfacing using the Imaris 7.7.2 software (Bitplane, UK). Iso-surfaced volumes of 10 voxels or less were excluded from the data sets in order to avoid including artifacts from general noise. In this case, 10 voxels translates into a spherical object with a diameter of ≤50 μm (The diameter of a β-cell is ≈10 μm). Lesion volumes were manually selected and pseudocolored in the tomographic images using Adobe Photoshop CS6 (Adobe, USA). The volumes were then quantified by 3D iso-surfacing using Imaris 7.7.2 (Bitplane, UK).

### Ultramicroscopy imaging

*ob*/*ob* mice at 26 weeks were anesthetized by intraperitoneal injection of a cocktail containing ¼ Hypnorm (VetaPharma Ltd., UK), ¼ Midazolam 5 mg/ml (Hameln pharma plus gmbh, Germany), ½ sterile water at a dose of 10 μl/g bodyweight and cardially perfused with wheat germ agglutinin lectin (Invitrogen W11262, 30 μg/g body weight) followed by perfusion with 4% paraformaldehyde (PFA). Pancreata were harvested and post-fixed in 4% PFA for 2 hours, washed in phosphate buffered saline (PBS), labeled for insulin (see below) and processed as for OPT imaging (see above). The samples were imaged by a LaVision biotech 2^nd^ generation UltraMicroscope (LaVision BioTec GmbH, Germany) and processed using Imaris7.7.2 (Bitplane, UK) or Drishti (v2.3 Australian National University, ANUSF VizLab, Canberra, ACT, Australia).

### Immunohistochemistry

Isolated pancreata were fixed in 4% PFA for 2 hours and washed in PBS. Pancreatic tissue were kept in 30% sucrose overnight and frozen in Optimal Cutting Temperature (OCT, Tissue-Tek 4583). Pancreata for paraffin sections were fixed in formalin overnight and processed for paraffin embedding. Tissue sections were stained and images were captured in a Nikon Eclipse E800 microscope (Nikon). Primary antibodies used were Guinea Pig anti insulin (DAKO A0564 1:500), Rabbit anti-fibrinogen (Abcam ab34269 1:500), Rabbit anti von willbrand factor (DAKO P0226 1:200), Rabbit anti fibronectin (Rockland 600-401-117-0.1 1:250), Rabbit anti Ki67 (Abcam 15580 1:500), Rabbit anti cleaved caspase 3 (Cell Signaling Technology 9661 1:500), Rabbit anti TGFβ1 (Novus Biologicals NBP1-03276 1:200), Mouse alpha smooth muscle actin ASMA-FITC conjugated (Abcam ab8211-100 1:400) and secondary antibodies used were goat Alexa 594 anti-rabbit (Molecular Probes A11012 1:500), goat Alexa 488 anti-Guinea pig (Molecular Probes A11073 1:500) and Goat Alexa 488 anti-rabbit (Molecular Probes A11034)).

### Proliferation analysis

The percentage of Ki67 positive cells was analyzed by counting Ki67^+^DAPI^+^-positive nuclei divided by the total number of DAPI-stained nuclei in Insulin^+^ cells. Images were collected using a Nikon E800 Fluorescence microscope (Nikon, Japan) and the images were analyzed manually using the counting tool in Photoshop CS5 Extended (version 12.0 × 64, Adobe, USA). 9454 and 9269 cells were counted for *ob*/*ob* and +/*?* mice respectively.

### Statistical Analysis

All the statistical analyses were executed in Microsoft Excel 2013. All values are shown as ± SEM. Student t test (two tailed) with a p-value < 0.05 was considered significant. R^2^ scores were calculated by creating a linear regression line.

## Additional Information

**How to cite this article**: Parween, S. *et al*. Intra-islet lesions and lobular variations in β-cell mass expansion in *ob/ob* mice revealed by 3D imaging of intact pancreas. *Sci. Rep.* **6**, 34885; doi: 10.1038/srep34885 (2016).

## Supplementary Material

Supplementary Information

Supplementary Movie 1

Supplementary Movie 2

Supplementary Movie 3
