# Supplementary material for: Intra-islet lesions and lobular variations in β-cell mass expansion in ob/ob mice revealed by 3D imaging of intact pancreas
Source: Sci Rep. 2016 Oct 7;6:34885. doi: 10.1038/srep34885 (PMC5054357; doi:10.1038/srep34885)

Supplementary Information

Intra-islet lesions and lobular variations in β-cell mass expansion in *ob/ob* mice revealed by 3D imaging of intact pancreas.

Saba Parween[1], Elena Kostromina[1], Christoffer Nord[1], Maria Eriksson[1], Per Lindström[3], Ulf Ahlgren[1*]

[1]Umeå Centre for Molecular Medicine, Umeå University, Umeå, Sweden
[2]Integrative Medical Biology, Umeå University, Umeå, Sweden
*Correspondence to ulf.ahlgren@umu.se

Supplementary Figure legends.

Supplementary figure 1. OPT images off β-cell mass distribution in the duodenal pancreatic lobe of *ob/ob* and lean control mice. Iso-surface rendered OPT images of representative duodenal lobes (same animals as in **Fig. 1A-J**) of lean control (**A**-**E**) and *ob/ob* (**F-J**) pancreata. The islet β-cell volumes are reconstructed based on the signal from insulin specific antibody staining (red) and pancreas outline (gray) is based on the signal from tissue autoflourescense. In contrast to lean controls, the expected expansion in BCV is clearly observed in *ob/ob* pancreata. Scale bar in (**J**) corresponds to 2 mm in (**A-J**).

Supplementary figure 2. OPT images of β-cell mass distribution in the gastric pancreatic lobe of *ob/ob* and lean control mice. Iso-surface rendered OPT images of representative gastric lobes (same animals as in **Fig. 1A-J**) of lean control (**A**-**E**) and *ob/ob* (**F-J**) pancreata. The islet β-cell volumes are reconstructed based on the signal from insulin specific antibody staining (red) and pancreas outline (gray) is based on the signal from tissue autoflourescense. In contrast to lean controls, the expected expansion in BCV is clearly observed in *ob/ob* pancreata. Scale bar in (**J**) corresponds to 2 mm in (**A-J**).

Supplementary figure 3. Proliferation analysis of *ob/ob* and lean control pancreata. A-F, Photomicrographs of pancreatic tissue sections from lean control (**A-C**) and *ob/ob* (**D-F**) pancreata at 52 weeks of age labeled for proliferation marker

Ki67 (red, **A** and **D**) and Insulin (**B** and **E**). Islets are delineated by a broken line. Prominent β-cell proliferation could be detected in the *ob/ob* islets at this stage. Note, the cytoplasmic staining in the periphery of the islet is unspecific binding in glucagon cells, characteristic of the utilized Ki67 antibody. **G**, β-cell proliferation determined in *ob/ob* and lean control mice at 52 weeks of age by immunofluorescence staining for insulin and Ki67. Values are given as ± SEM (n=4 for *+/?* and n=3 for o*b/ob*). p=0.34. Scale bar in (**F**) corresponds to 50µm in (**A-F**).

Supplementary figure 4. Immunohistochemical assessment of apoptosis and fibrosis in *ob/ob* islets. **A-F**, Photomicrographs of representative pancreatic cryo sections from lean control (**A-C**) and *ob/ob* (**D-F**) pancreata at 26 weeks of age labeled for apoptosis marker cleaved Caspase 3 (**A, D**) and DAPI (**B, E**). Islets are enclosed by a broken white line and cystic lesions by a broken yellow line. There are no sign of increased apoptosis in the area of the lesions. **G-H**, Section of duodenal epithelium from a developing embryo of C57BL/6 mouse included as a positive control for the Cleaved Caspase3 antibody labelings. **J-K**, Photomicrographs of representative pancreatic tissue sections from lean control (**J-L**) and *ob/ob* (**M-O**) pancreata at 26 weeks of age labeled for TGF-β (Red, **J** and **K**) as a marker of fibrosis and Smooth muscle α-actin as a marker for large blood vessels. (Green, **K** and **N**). TGF-β1 expression is confined to Smooth muscle α-actin positive areas and could not be detected within the lesions. Scale bar in (**O**) is 50µm in (**A-O**).

Supplementary Figure 5. Weight and blood glucose levels of the investigated animals. **A**, Average body weights of the animals utilized in the study in conjunction with organ isolation. **B**, Average blood glucose levels of the animals utilized in the study in conjunction with organ isolation. Open circles represent Lean control animals and closed circles represent ob/ob animals. Data is shown as means ± SEM (n=5) where *P < 0.05; **P < 0.01 and ***P < 0.001.

Supplementary Movie captions

Supplementary Movie 1. Movie of tomographic Z-stack displaying cystic lesions in hypertrophic islets of *ob/ob* pancreata. Movie showing tomographic images of a *ob/ob* pancreas (splenic lobe at 26 weeks of age). This signal from insulin labeled islets is pseudocolored (red) and the the tomographic reconstruction of the exocrine parenchyma appear as (dark grey).

Supplementary Movie 2. Movie illustrating the 3D distribution of islets lesions in the *ob/ob* pancreas. The islet β-cell volumes are reconstructed based on the signal from insulin specific antibody staining (red) and the outlines of the cystic lesions were manually delineated in the tomographic sections (gray). The outline of the organ is based on the signal from tissue autofluorescence (dark gray). The specimen is the same as seen in **Fig. 1S** and **Fig. 4A-D**.

Supplementary Movie 3. Movie illustrating that islet lesions do not represent enlarged vessels. The movie shows an ultramicroscopy Z-stack through an *ob/ob* pancreas (duodenal lobe, 26 weeks of age) labeled for Insulin (red) and wheat germ agglutinin lectin (green). Note that wheat germ agglutinin lectin does not label the inner lining of the lesion.

Figure S1.

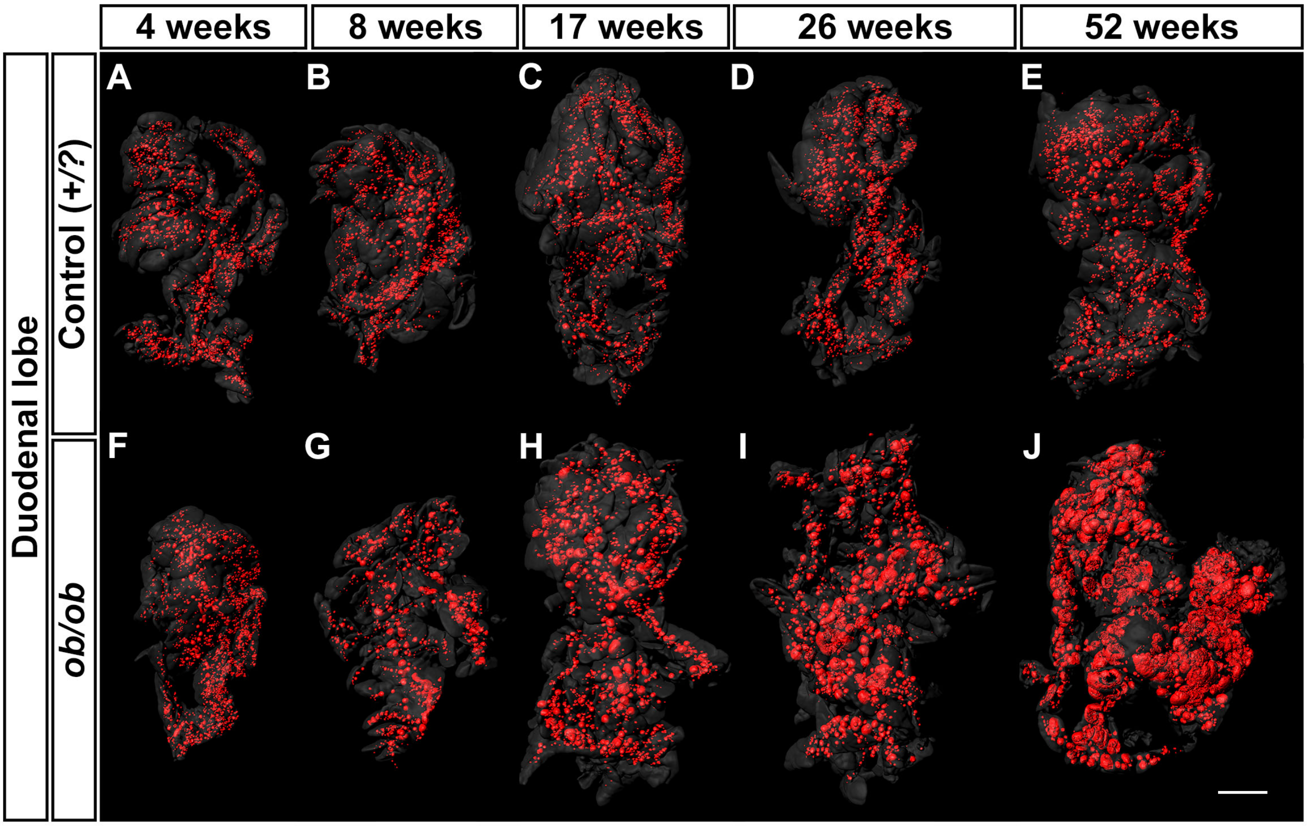

4 weeks
8 weeks
17 weeks
26 weeks
52 weeks
Duodenal lobe
Control (+/?)
ob/ob
A
B
C
D
E
F
G
H
I
J

Figure S2.

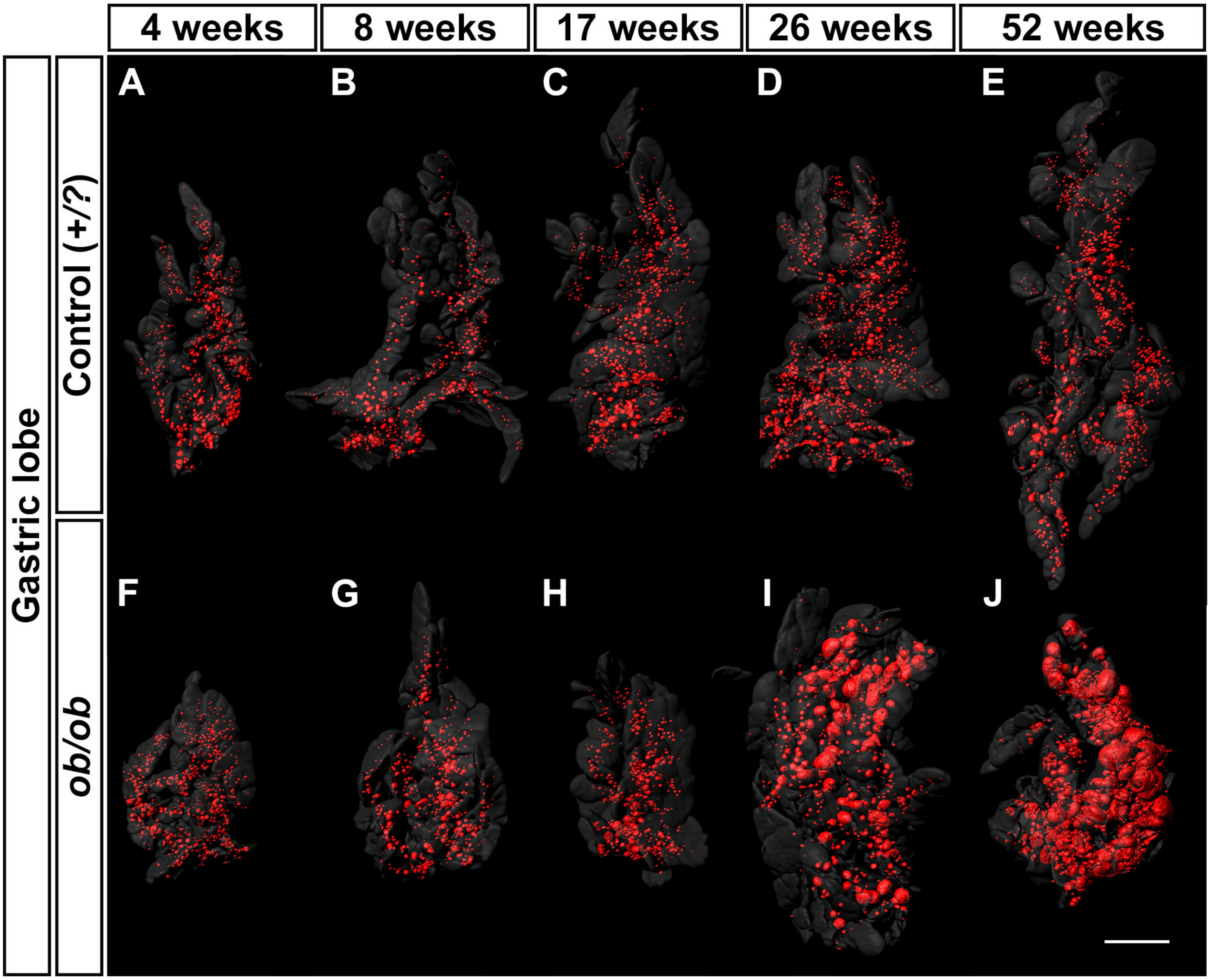

4 weeks
8 weeks
17 weeks
26 weeks
52 weeks
Gastric lobe
Control (+/?)
ob/ob
A
B
C
D
E
F
G
H
I
J

Figure-S3 (Ahlgren)

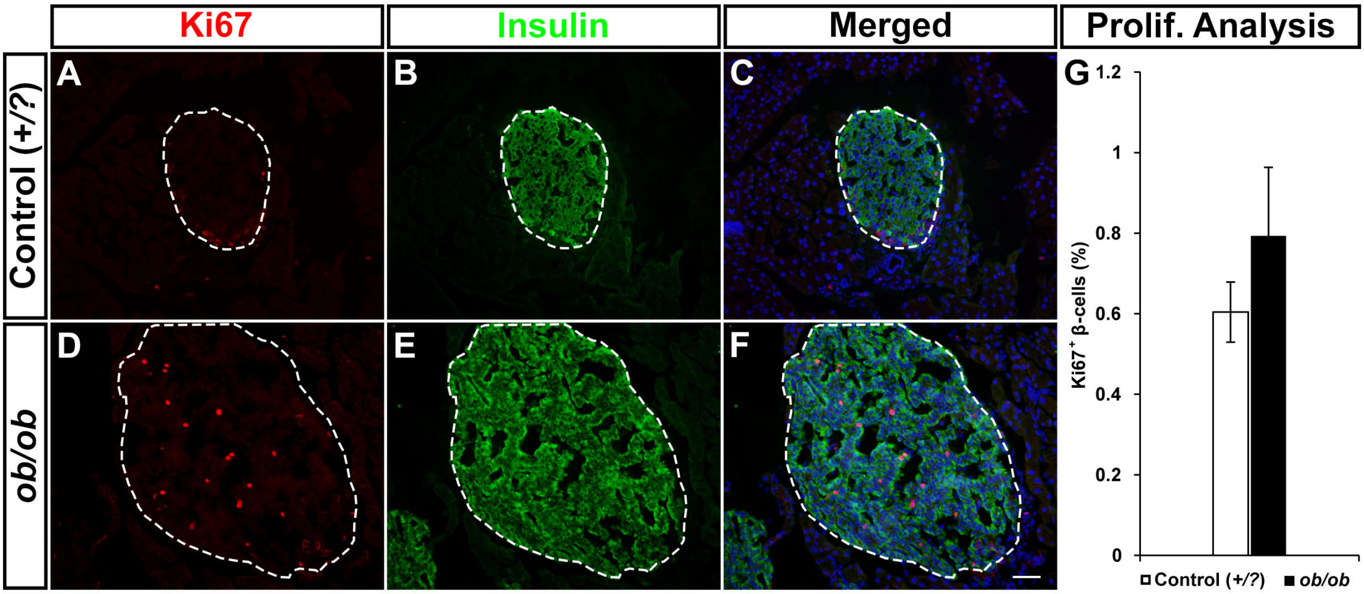

Figure-S4 (Ahlgren)

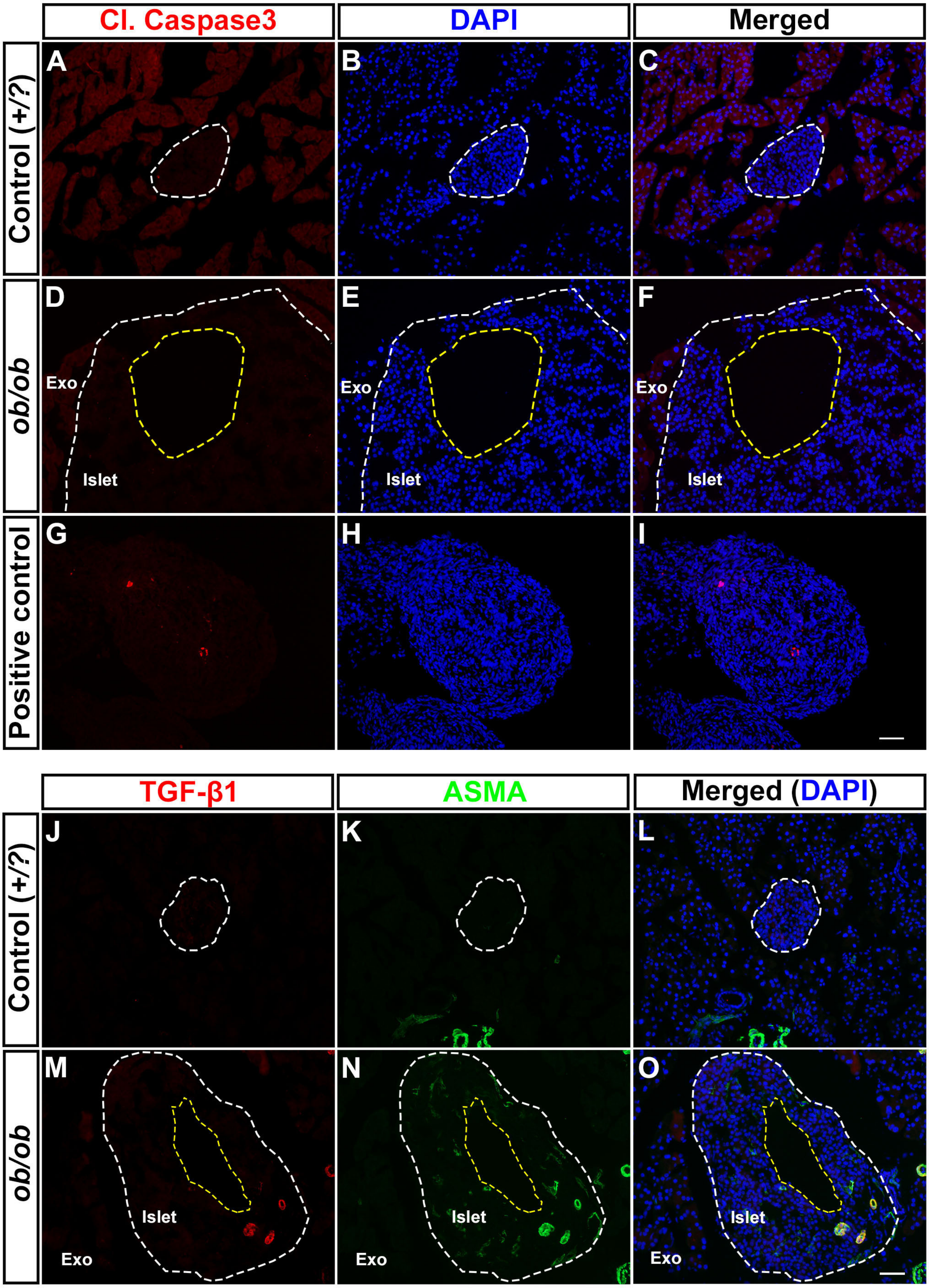

Figure-S5 (Ahlgren)

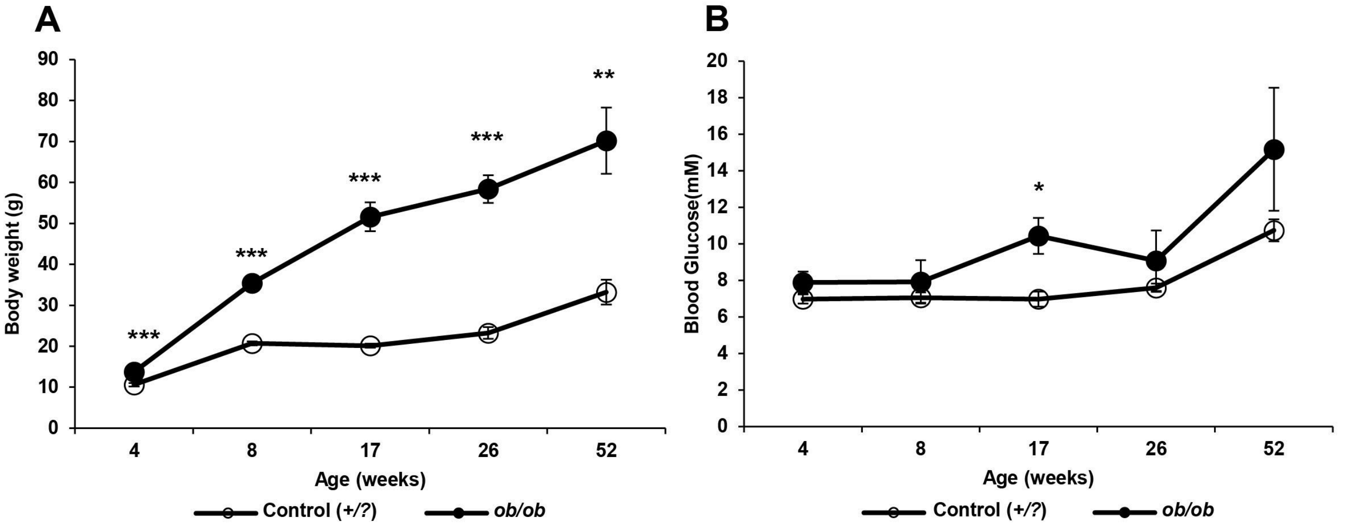

Supplement: Supplementary Information [file srep34885-s1.pdf]
